# Supplementary material for: Antifungal Activity of Soft Tissue Extract from the Garden Snail Helix aspersa (Gastropoda, Mollusca)
Source: Molecules. 2022 May 16;27(10):3170. doi: 10.3390/molecules27103170 (PMC9144933; doi:10.3390/molecules27103170)
Supplement: Supplementary file 1 [file molecules-27-03170-s001.zip › molecules-1701728-supplementary.pdf]

molecules

MDPI

Supplmentary information

Antifungal Activity of Soft Tissue Extract from the Garden Snail *Helix Aspersa* (Gastropoda, Mollusca)

Hoda H. Abd-El Azeem [1,*], Gamalat Y. Osman [1], Hesham R. El-Seedi [2,3,4,5,*], Ahmed M Fallatah [6], Shaden A. M. Khalifa [7] and Mohamed M. Gharib [8]

1 Department of Zoology, Faculty of Sciences, Menoufia University, 32512 Shebin El-Kom, Egypt; hodaelsheikh483@gmail.com (H.H.A.-E.A); and dr.gyosman@yahoo.com (G.Y.O)
2 Pharmacognosy Group, Department of Pharmaceutical Biosciences, Biomedical Centre, Uppsala University, Uppsala, Box 591, SE 751 24, Sweden; hesham.el-seedi@farmbio.uu.se
3 International Research Center for Food Nutrition and Safety, Jiangsu University, Zhenjiang 212013, China
4 International Joint Research Laboratory of Intelligent Agriculture and Agri-products Processing (Jiangsu University), Jiangsu Education Department, China
5 Department of Chemistry, Faculty of Science, Menoufia University, 32512 Shebin El-Kom, Egypt
6 Department of Chemistry, College of Science, Taif University, P.O. Box 11099, Taif 21944, Saudi Arabia; a.fallatah@tu.edu.sa
7 Department of Molecular Biosciences, Stockholm University, the Wenner-Gren Institute, SE-106 91 Stockholm, Sweden; shaden.khalifa.2014@gmail.com
8 Department of Botany, Faculty of Sciences, Menoufia University, 32512 Shebin El-Kom, Egypt; gharieb2000@yahoo.com

*Correspondence: hodaelsheikh483@gmail.com (H.H.A.-E.A); .hesham.el-seedi@farmbio.uu.se (H.R.E.-S)

Citation: Abd-El Azeem, H.H.; Osman, G.Y.; El-Seedi, H.R.; Fallatah, A.M.; Khalifa, S.A.M.; Gharib, M.M. Antifungal Activity of Soft Tissue Extract from the Garden Snail *Helix Aspersa* (Gastropoda, Mollusca). *Molecules* **2022**, *27*, 3170. https://doi.org/10.3390/molecules27103170

Academic Editor: Jolanta Mierzejewska

Received: 12 April 2022
Accepted: 11 May 2022
Published: 16 May 2022

Publisher's Note: MDPI stays neutral with regard to jurisdictional claims in published maps and institutional affiliations.

Copyright: © 2022 by the authors. Licensee MDPI, Basel, Switzerland. This article is an open access article distributed under the terms and conditions of the Creative Commons Attribution (CC BY) license (https://creativecommons.org/licenses/by/4.0/).

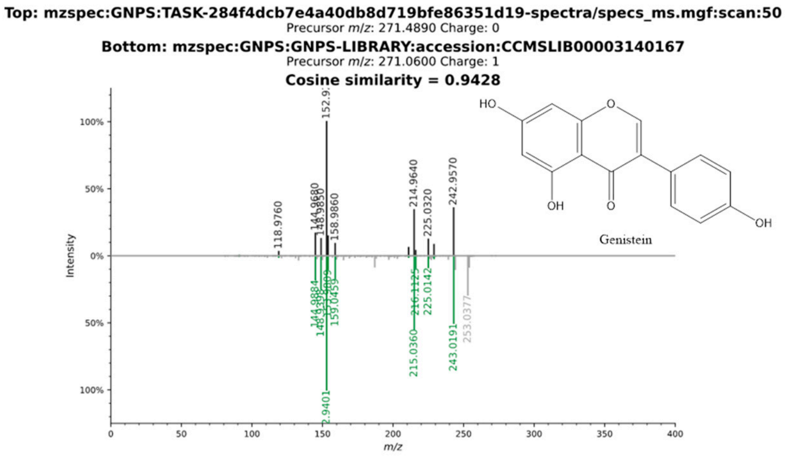

a

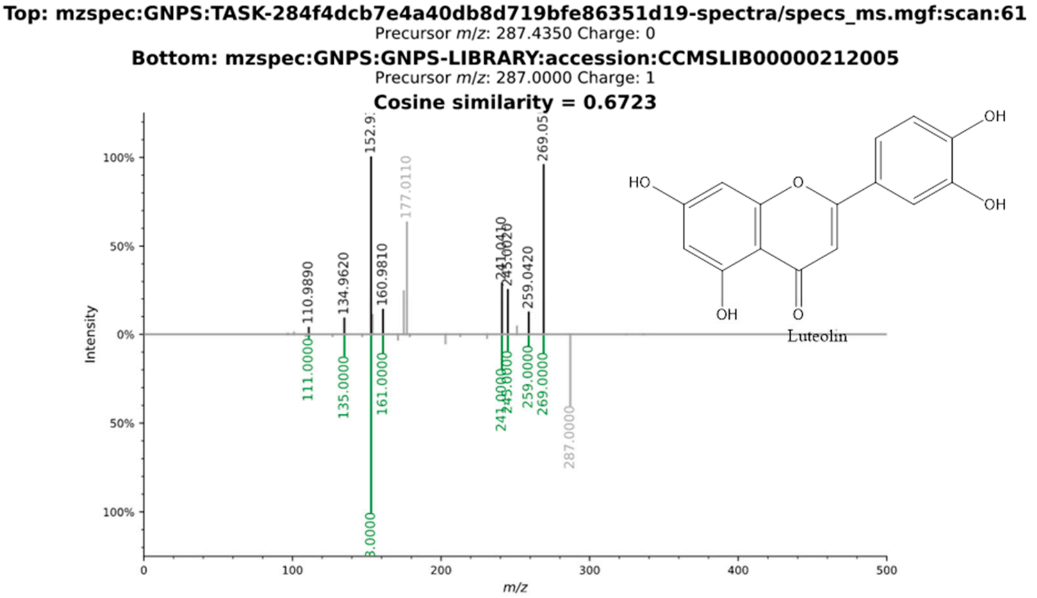
Top: mzspec:GNPS:TASK-284f4dcb7e4a40db8d719bfe86351d19-spectra/specs_ms.mgf:scan:61
Precursor m/z: 287.4350 Charge: 0
Bottom: mzspec:GNPS:GNPS-LIBRARY:accession:CCMSLIB00000212005
Precursor m/z: 287.0000 Charge: 1
Cosine similarity = 0.6723
Intensity
m/z
Luteolin
HO
OH
OH
OH
O
O

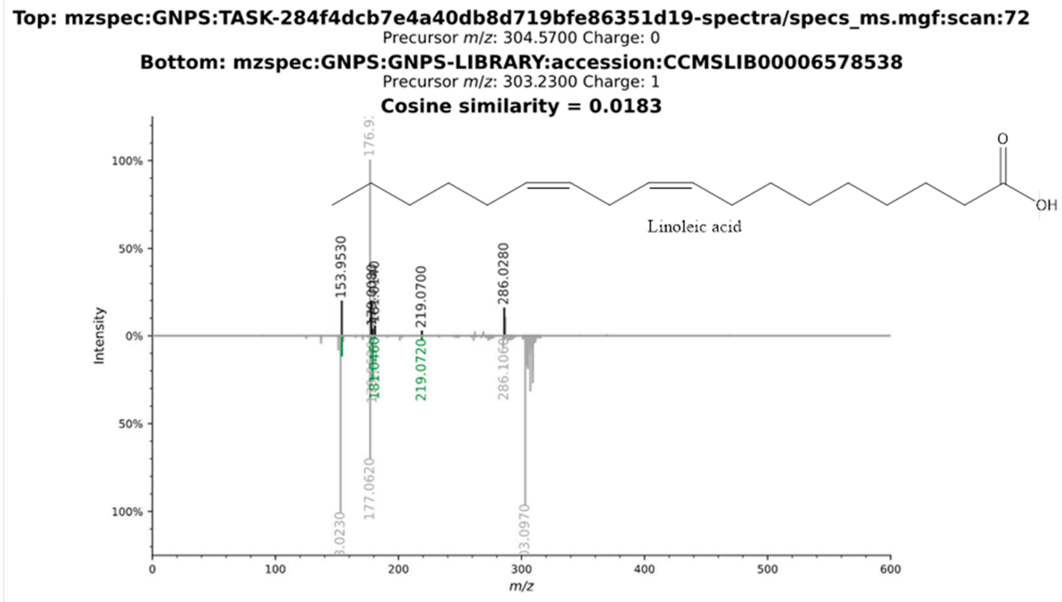
Top: mzspec:GNPS:TASK-284f4dcb7e4a40db8d719bfe86351d19-spectra/specs_ms.mgf:scan:72
Precursor m/z: 304.5700 Charge: 0
Bottom: mzspec:GNPS:GNPS-LIBRARY:accession:CCMSLIB00006578538
Precursor m/z: 303.2300 Charge: 1
Cosine similarity = 0.0183
Intensity
m/z
Linoleic acid
O
OH

b

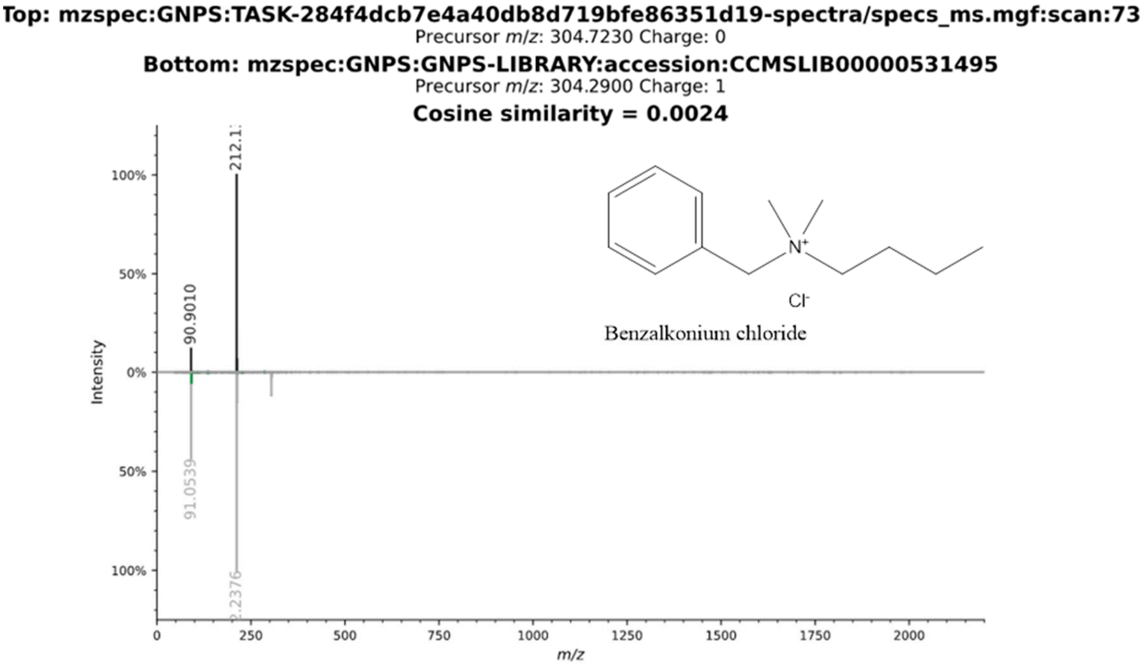
Top: mzspec:GNPS:TASK-284f4dcb7e4a40db8d719bfe86351d19-spectra/specs_ms.mgf:scan:73
Precursor m/z: 304.7230 Charge: 0
Bottom: mzspec:GNPS:GNPS-LIBRARY:accession:CCMSLIB00000531495
Precursor m/z: 304.2900 Charge: 1
Cosine similarity = 0.0024
90.9010
91.0539
Intensity
m/z
Cl⁻
Benzalkonium chloride

c

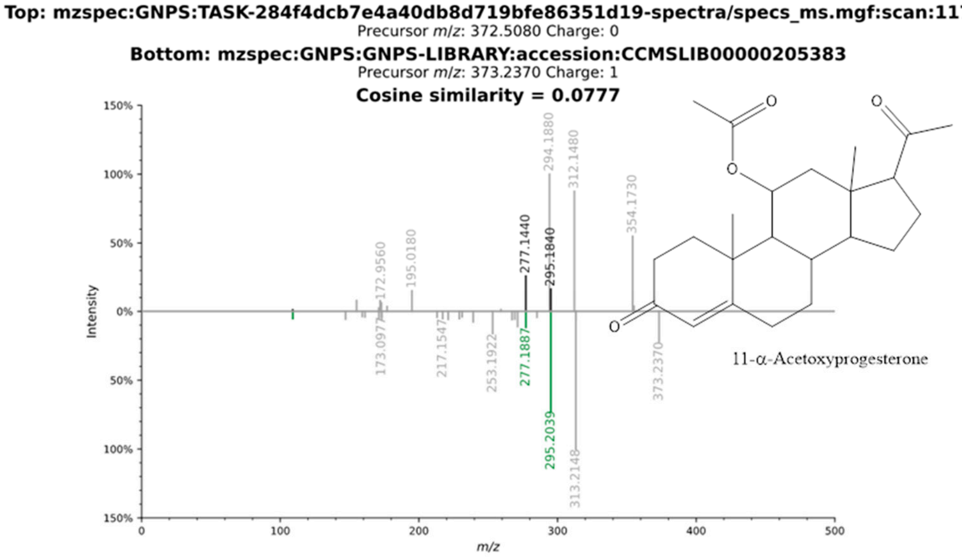
Top: mzspec:GNPS:TASK-284f4dcb7e4a40db8d719bfe86351d19-spectra/specs_ms.mgf:scan:117
Precursor m/z: 372.5080 Charge: 0
Bottom: mzspec:GNPS:GNPS-LIBRARY:accession:CCMSLIB00000205383
Precursor m/z: 373.2370 Charge: 1
Cosine similarity = 0.0777
172.9560
195.0180
277.1440
295.1840
294.1880
312.1480
354.1730
217.1547
253.1922
277.1887
295.2039
313.2148
373.2370
Intensity
m/z
11-α-Acetoxyprogesterone

d

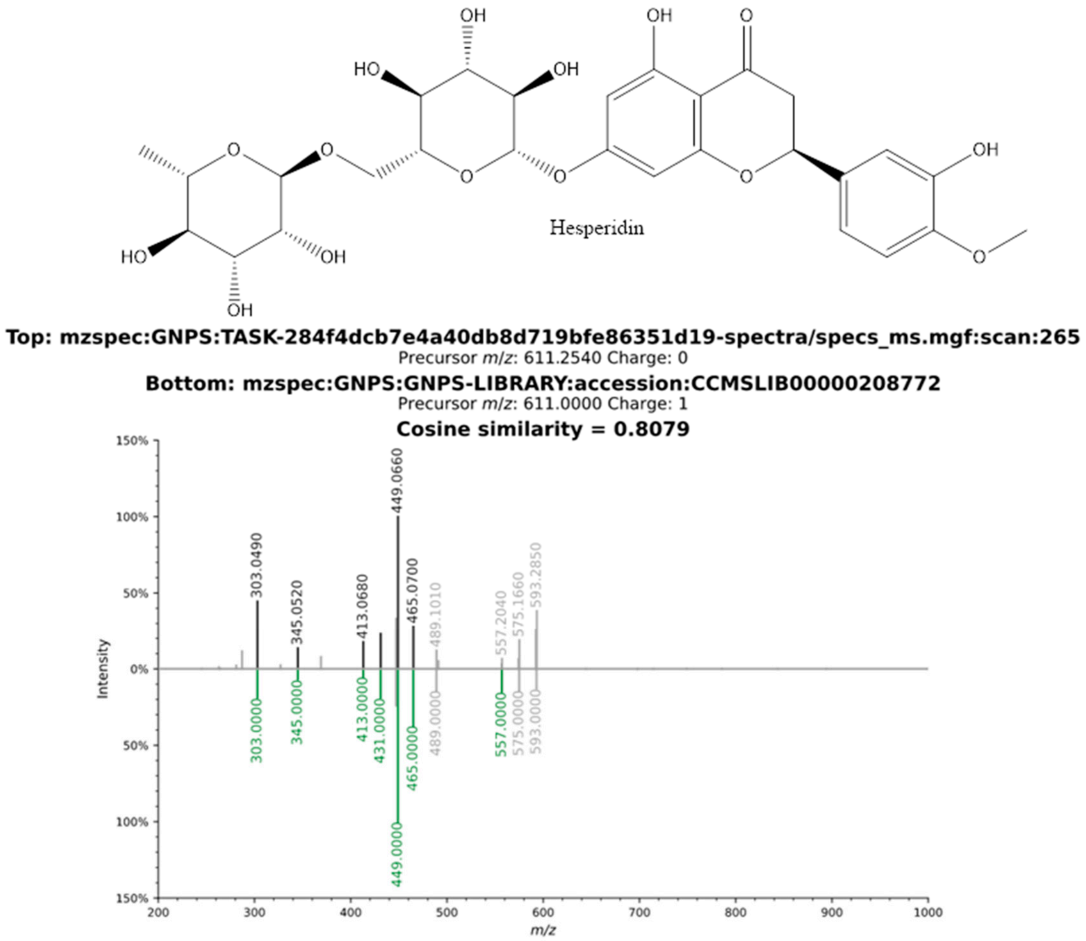

e

Figure S1. The identified metabolites' parent masses and fragments of the methanol and acetone viscera extracts of the Helix aspersa from the raw mass spectrum; (a) genistein; (b) luteolin; (c) linoleic acid; (d)11-alpha-acetoxyprogesterone and (e) hesperidin
